# Supplementary material for: Growth of Neogene Andes linked to changes in plate convergence using high-resolution kinematic models
Source: Nat Commun. 2022 Mar 15;13:1339. doi: 10.1038/s41467-022-29055-4 (PMC8924272; doi:10.1038/s41467-022-29055-4)
Supplement: Supplementary file 1 — Supplementary Information [file 41467_2022_29055_MOESM1_ESM.pdf]

# Supplementary Information

**Supplementary References to Figure 3.**
Panels D to G of Figure 3 where constructed based on a compilation and analysis of different sources informing about the tectonomagmatic evolution of the Andean margin at the studied latitudes. Here we provide details of literature sources used for each panel:

- Fig. 3D: Stalder et al. (2020), Oncken et al. (2006 and 2012), Victor et al. (2004), Trumbull et al. (2006), Garzione et al. (2014), Jordan et al. (2010), Horton (2018).
- Fig. 3E: Giambiagi et al. (2015, 2016, 2017), Stalder et al. (2020), Lossada et al. (2017), Suriano et al. (2017), Rodriguez et al. (2018), Allmendinger and Judge (2014), Ramos et al. (2002), Litvak et al. (2018), Mardonez et al. (2020)
- Fig. 3F: Orts et al. (2012, 2015), Garcia-Morabito et al. (2011, 2012), Ramos et al. (2015), Echaurren et al. (2016), Horton (2018), Encinas et al. (2018 and 2021), Bechis et al. (2014), Cembrano et al. (2002), Fernández Paz et al. (2019), Navarrete et al. (2020).
- Fig. 3G: Oncken et al. (2012), Anderson et al. (2018), Trumbull et al. (2006), Garzione et al. (2014 and 2017)
- Fig. 3H: Allmendinger and Judge (2014), Mardonez et al. (2020).

All these references are listed at the end of this document.

## Supplementary Figures

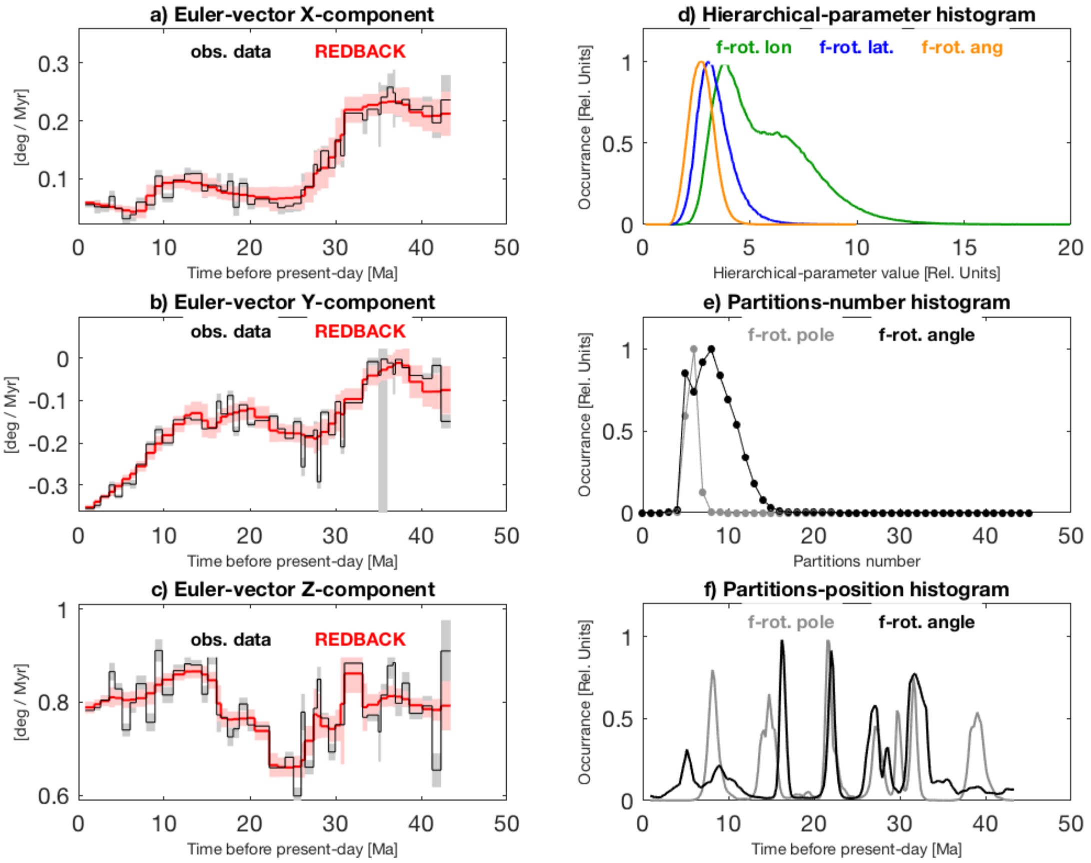


Supplementary Figure 1: Diagnostics for the quality of noise mitigation of Redback applied to the finite rotations of Croon et al. (2008), which reconstruct the past position of the Pacific plate relative to the Antarctica plate. See Iaffaldano et al. (2014) and the Redback user manual at http://www.iearth.org.au/codes/REDBACK/ for more details.

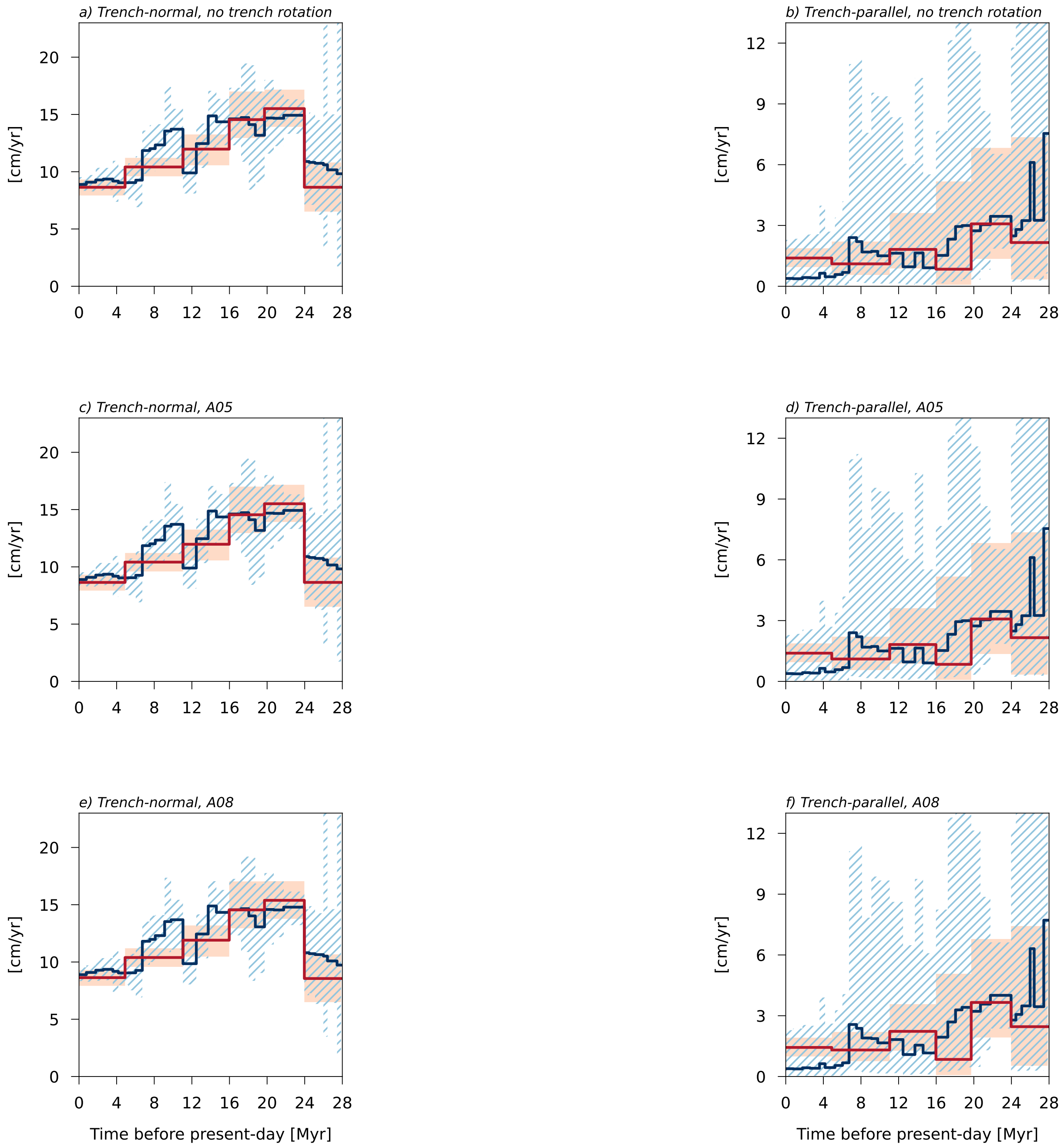


Supplementary Figure 2A: Trench-normal (left) and trench-parallel (right) component of convergence between Nazca and South America computed from our model (in blue) and Somoza and Ghidella, 2012 (in red) at 20°S considering no rotation of the trench axis over time (upper panel), rotations as reported by Allmendinger et al. (2005, middle panel – A05) and Arriagada et al. (2008, lower panel – A08). Lines are the average (nominal) value and shaded/hatched areas show 95% confidence.

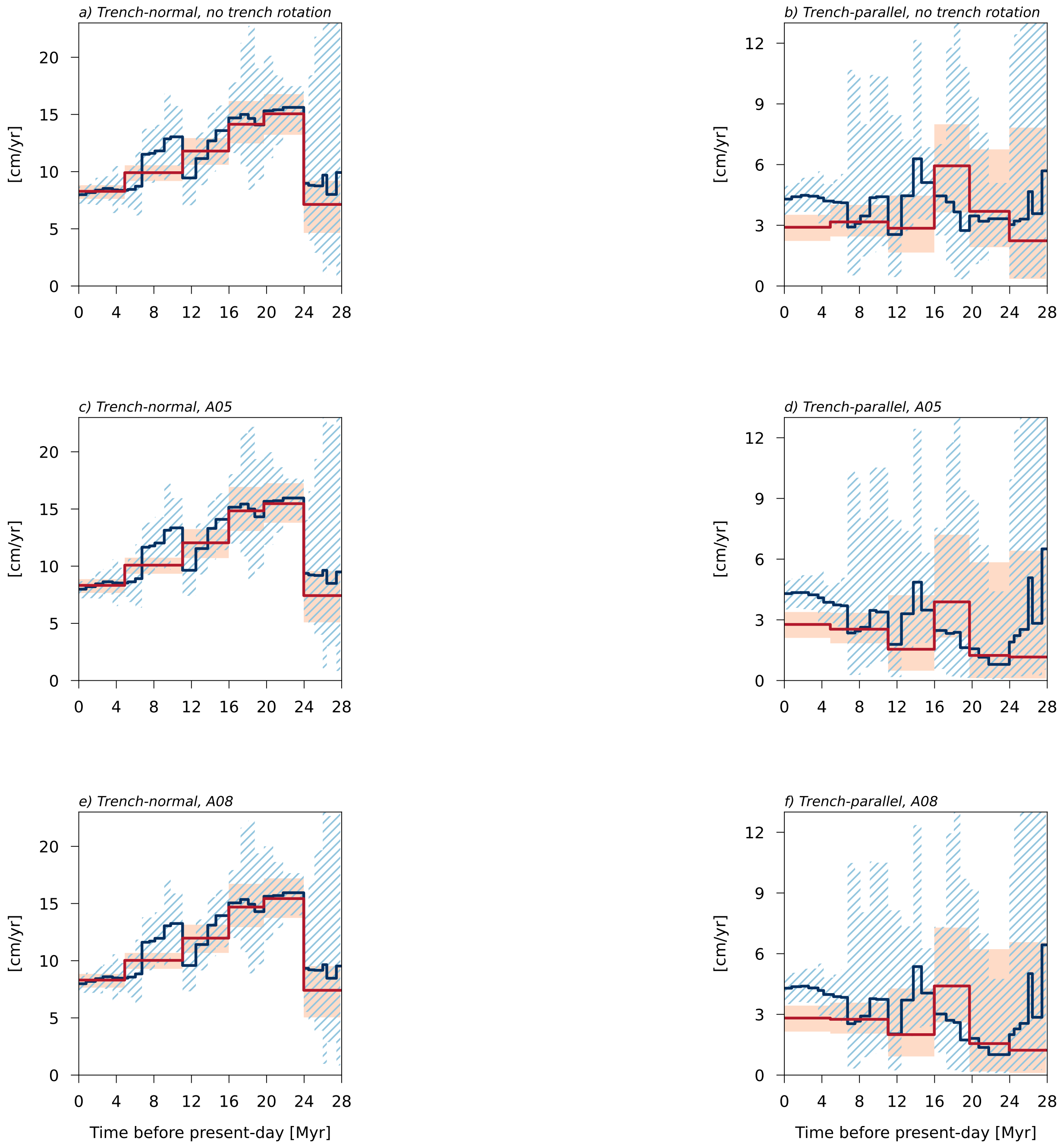


Supplementary Figure 2B: Same as Supplementary Figure 2A, but at 30°S.

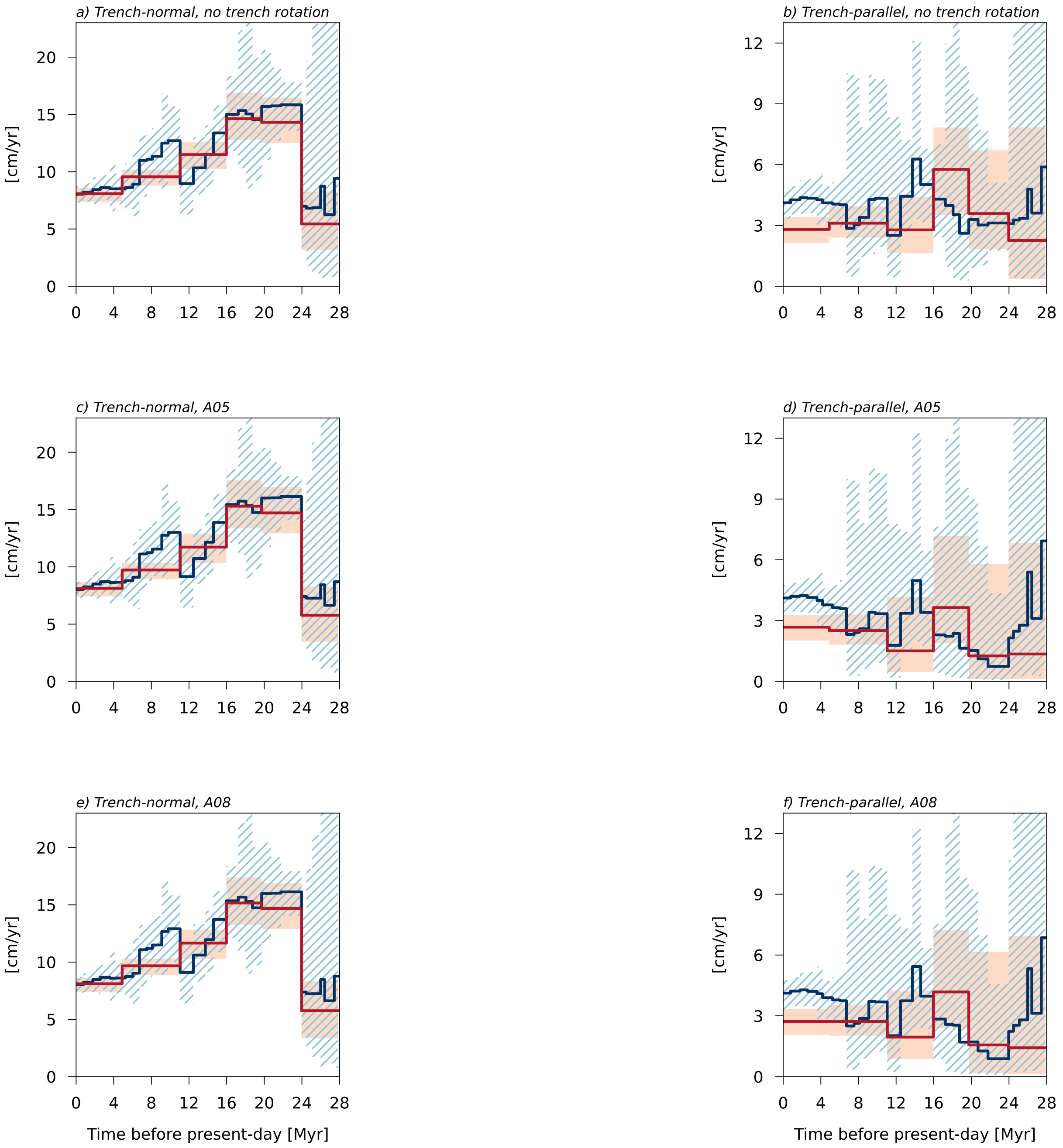


Supplementary Figure 2C: Same as Supplementary Figure 2A, but at 40°S.

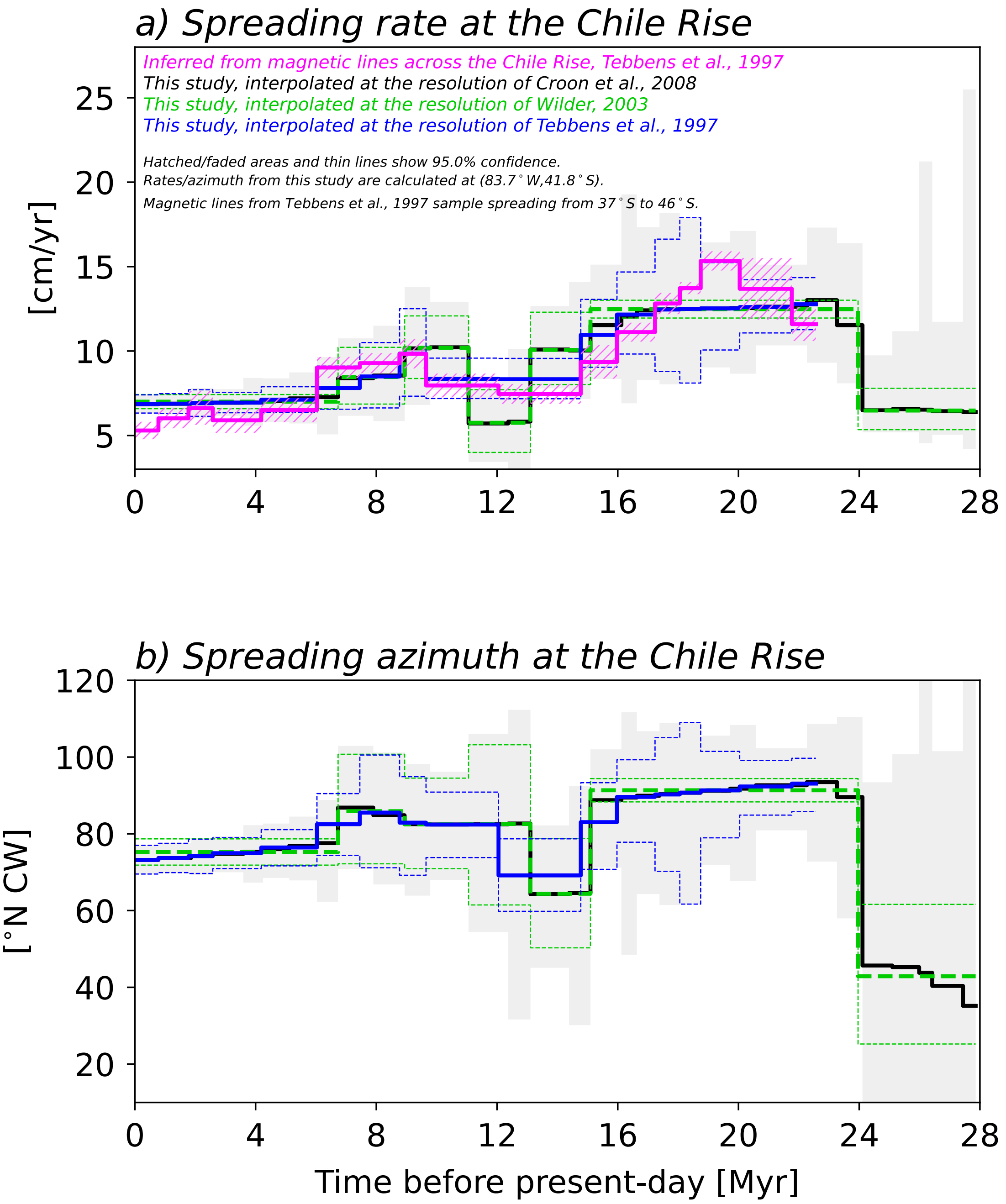


Supplementary Figure 3: Spreading rate (a) and azimuth (b) of the Chile Rise. Rates and azimuth from our reconstruction, which follows the Nazca – Pacific – Antarctica circuit, are calculated at the indicated position. We utilize three alternative temporal resolutions, in addition to that shown in the main-text figures. They are i) the resolution of the Nazca – Pacific finite rotations of Wilder (2003), ii) that of the Pacific – Antarctica finite rotations of Croon et al. (2008), and iii) that of the Chile Rise spreading-rate history inferred by Tebbens et al. (1997) from 17 magnetic lines directly observed across the Chile Rise, between 37° S and 46° S (see Table 2 in their study). The facts that the pattern of deceleration and acceleration coinciding with the Quechua deformation phase remains visible regardless of the utilized resolution. The agreement with the independently-established spreading-rate history of Tebbens et al. (1997) indicates that such kinematic pattern is a true feature of our reconstruction, rather than an artifact due to finite-rotations interpolation.

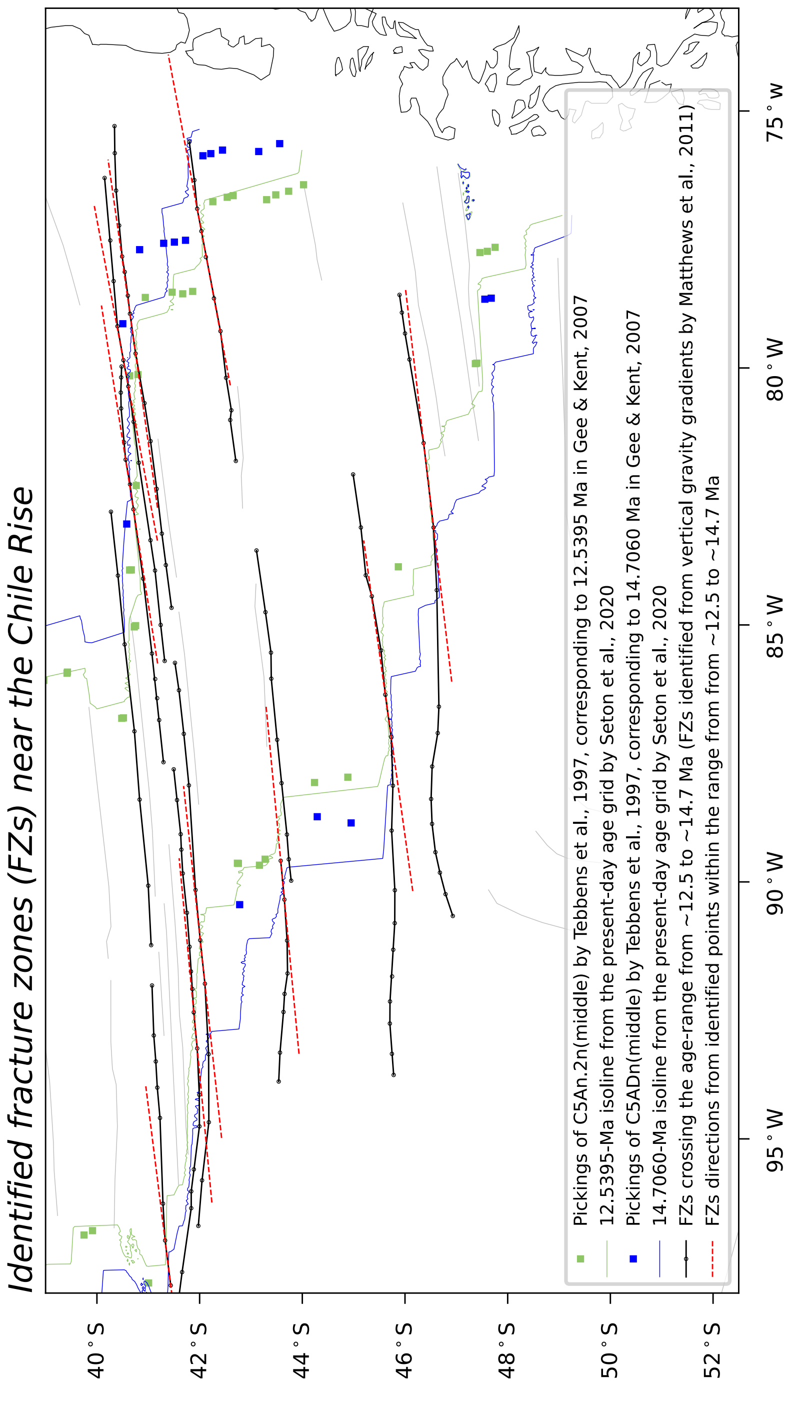


Supplementary Figure 4: Visual analysis of fracture zones (FZs) near the Chile Rise. In black are FZs mapped from vertical gravity gradients (see Matthews et al., 2011 for more details) and publicly available at https://www.soest.hawaii.edu/PT/GSFML/. Green/Blue squares are magnetic pickings of anomaly C5An.2n (middle)/C5AD (middle) by Tebbens et al. (1997, publicly available at https://www.soest.hawaii.edu/PT/GSFML/), corresponding to 12.5395 and 14.7060 Ma according to the geomagnetic reversal timescale of Gee and Kent (2007). Green/Blue lines are isoage lines (corresponding to the same-color-squares ages) from the present-day ocean-floor age map of Seton et al. (2020). Magnetic pickings and isoage lines are utilized to identify the FZs points falling within the age range from ~14 to ~12 Ma. These points are then used to trace the spreading azimuth (in dashed red) during that time period.

**Supplementary References**


- Allmendinger, R. W., Smalley Jr, R., Bevis, M., Caprio, H., & Brooks, B. (2005). Bending the Bolivian orocline in real time. *Geology*, *33*(11), 905-908.
- Allmendinger, R. W., & Judge, P. A. (2014). The Argentine Precordillera: A foreland thrust belt proximal to the subducted plate. *Geosphere*, *10*(6), 1203-1218.
- Arriagada, C., Roperch, P., Mpodozis, C., & Cobbold, P. R. (2008). Paleogene building of the Bolivian Orocline: Tectonic restoration of the central Andes in 2-D map view. *Tectonics*, *27*(6).
- Bechis, F., Encinas, A., Concheyro, A., Litvak, V. D., Aguirre-Urreta, B., & Ramos, V. A. (2014). New age constraints for the Cenozoic marine transgressions of northwestern Patagonia, Argentina (41–43 S): Paleogeographic and tectonic implications. *Journal of South American Earth Sciences*, *52*, 72-93.
- Cembrano, J., Lavenu, A., Reynolds, P., Arancibia, G., López, G., & Sanhueza, A. (2002). Late Cenozoic transpressional ductile deformation north of the Nazca–South America–Antarctica triple junction. *Tectonophysics*, *354*(3-4), 289-314.
- Croon, M.B, Cande, S.C., & Stoick, J.M., 2008, Revised Pacific-Antarctic plate motions and geophysics of the Menard Fracture Zone, *Geochem. Geophys. Geosys.*, 9, Q07001.
- Echaurren, A., Folguera, A., Gianni, G., Orts, D., Tassara, A., Encinas, A., ... & Valencia, V. (2016). Tectonic evolution of the North Patagonian Andes (41–44 S) through recognition of syntectonic strata. *Tectonophysics*, *677*, 99-114.
- Encinas, A., Folguera, A., Bechis, F., Finger, K. L., Zambrano, P., Pérez, F., ... & Orts, D. (2018). The late Oligocene–early Miocene marine transgression of Patagonia. In *The Evolution of the Chilean-Argentinean Andes* (pp. 443-474). Springer, Cham.
- Encinas, A., Sagripanti, L., Rodríguez, M. P., Orts, D., Anavalón, A., Giroux, P., ... & Valencia, V. (2020). Tectonosedimentary evolution of the Coastal Cordillera and Central Depression of south-Central Chile (36° 30′-42° S). *Earth-Science Reviews*, 103465.
- Fernández Paz, L., Bechis, F., Litvak, V. D., Echaurren, A., Encinas, A., González, J., ... & Folguera, A. (2019). Constraints on trenchward arc migration and backarc magmatism in the north patagonian Andes in the context of Nazca plate rollback. *Tectonics*, *38*(11), 3794-3817.
- Garcia-Morabito, E. G., & Ramos, V. A. (2012). Andean evolution of the Aluminé fold and thrust belt, Northern Patagonian Andes (38 30′–40 30′ S). *Journal of South American Earth Sciences*, *38*, 13-30.
- Garcia-Morabito, E. G., Götze, H. J., & Ramos, V. A. (2011). Tertiary tectonics of the Patagonian Andes retro-arc area between 38 15′ and 40 S latitude. *Tectonophysics*, *499*(1-4), 1-21.
- Garzione, C. N., Auerbach, D. J., Smith, J. J. S., Rosario, J. J., Passey, B. H., Jordan, T. E., & Eiler, J. M. (2014). Clumped isotope evidence for diachronous surface cooling of the Altiplano and pulsed surface uplift of the Central Andes. *Earth and Planetary Science Letters*, *393*, 173-181.
- Garzione, C. N., McQuarrie, N., Perez, N. D., Ehlers, T. A., Beck, S. L., Kar, N., ... & Lease, R. O. (2017). Tectonic evolution of the Central Andean plateau and implications for the growth of plateaus. *Annual Review of Earth and Planetary Sciences*, *45*, 529-559.
- Garzione, C. N., Hoke, G. D., Libarkin, J. C., Withers, S., MacFadden, B., Eiler, J., ... & Mulch, A. (2008). Rise of the Andes. *science*, *320*(5881), 1304-1307.
- Giambiagi, L., Tassara, A., Mescua, J., Tunik, M., Alvarez, P. P., Godoy, E., ... & Tapia, F. (2015). Evolution of shallow and deep structures along the Maipo–Tunuyán transect (33°

40′ S): from the Pacific coast to the Andean foreland. *Geological Society, London, Special Publications*, *399*(1), 63-82.
- Giambiagi, L., Mescua, J., Bechis, F., Hoke, G., Suriano, J., Spagnotto, S., ... & Folguera, A. (2016). Cenozoic orogenic evolution of the southern central Andes (32–36 S). In *Growth of the Southern Andes* (pp. 63-98). Springer, Cham.
- Giambiagi, L., Álvarez, P. P., Creixell, C., Mardonez, D., Murillo, I., Velásquez, R., ... & Barrionuevo, M. (2017). Cenozoic Shift From Compression to Strike-Slip Stress Regime in the High Andes at 30° S, During the Shallowing of the Slab: Implications for the El Indio/Tambo Mineral District. *Tectonics*, *36*(11), 2714-2735.
- Horton, B. K. (2018). Tectonic regimes of the central and southern Andes: Responses to variations in plate coupling during subduction. *Tectonics*, *37*(2), 402-429.
- Iaffaldano, G., Hawkins, R., Bodin, T. & Sambridge, M., 2014, REDBACK: open-source software for efficient noise-reduction in plate kinematic reconstructions, *Geochem. Geophys. Geosys.*, 15, pp. 1663-1670.
- Jordan, T. E., Nester, P. L., Blanco, N., Hoke, G. D., Dávila, F., & Tomlinson, A. J. (2010). Uplift of the Altiplano-Puna plateau: A view from the west. *Tectonics*, *29*(5).
- Litvak, V. D., Poma, S., Jones, R. E., Paz, L. F., Iannelli, S. B., Spagnuolo, M., ... & Ramos, V. A. (2018). The late Paleogene to Neogene volcanic arc in the southern central Andes (28–37 S). In *The Evolution of the Chilean-Argentinean Andes* (pp. 503-536). Springer, Cham.
- Lossada, A. C., Giambiagi, L., Hoke, G. D., Fitzgerald, P. G., Creixell, C., Murillo, I., ... & Suriano, J. (2017). Thermochronologic evidence for late Eocene Andean mountain building at 30 S. *Tectonics*, *36*(11), 2693-2713.
- Mardonez, D., Suriano, J., Giambiagi, L., Mescua, J., Lossada, A., Creixell, C., & Murillo, I. (2020). The Jáchal river cross-section revisited (Andes of Argentina, 30° S): Constraints from the chronology and geometry of neogene synorogenic deposits. *Journal of South American Earth Sciences*, *104*, 102838.
- Navarrete, C., Gianni, G., Massaferro, G., & Butler, K. (2020). The fate of the Farallon slab beneath Patagonia and its links to Cenozoic intraplate magmatism, marine transgressions and topographic uplift. *Earth-Science Reviews*, *210*, 103379.
- Oncken, O., Hindle, D., Kley, J., Elger, K., Victor, P., & Schemmann, K. (2006). Deformation of the central Andean upper plate system—Facts, fiction, and constraints for plateau models. In *The Andes* (pp. 3-27). Springer, Berlin, Heidelberg.
- Oncken, O., Boutelier, D., Dresen, G., & Schemmann, K. (2012). Strain accumulation controls failure of a plate boundary zone: Linking deformation of the Central Andes and lithosphere mechanics. *Geochemistry, Geophysics, Geosystems*, *13*(12).
- Orts, D. L., Folguera, A., Encinas, A., Ramos, M., Tobal, J., & Ramos, V. A. (2012). Tectonic development of the North Patagonian Andes and their related Miocene foreland basin (41° 30′-43° S). *Tectonics*, *31*(3).
- Orts, D. L., Folguera, A., Giménez, M., Ruiz, F., Vera, E. A. R., & Klinger, F. L. (2015). Cenozoic building and deformational processes in the North Patagonian Andes. *Journal of Geodynamics*, *86*, 26-41.
- Ramos, V. A., Cristallini, E. O., & Pérez, D. J. (2002). The Pampean flat-slab of the Central Andes. *Journal of South American earth sciences*, *15*(1), 59-78.
- Ramos, M. E., Tobal, J. E., Sagripanti, L., Folguera, A., Orts, D. L., Giménez, M., & Ramos, V. A. (2015). The North Patagonian orogenic front and related foreland evolution during the Miocene, analyzed from synorogenic sedimentation and U/Pb dating (∼ 42° S). *Journal of South American Earth Sciences*, *64*, 467-485.

- Rodríguez, M. P., Charrier, R., Brichau, S., Carretier, S., Farías, M., de Parseval, P., & Ketcham, R. A. (2018). Latitudinal and longitudinal patterns of exhumation in the Andes of north-central Chile. *Tectonics*, *37*(9), 2863-2886.
- Stalder, N. F., Herman, F., Fellin, G. M., Coutand, I., Aguilar, G., Reiners, P. W., & Fox, M. (2020). The relationships between tectonics, climate and exhumation in the Central Andes (18–36° S): Evidence from low-temperature thermochronology. *Earth-Science Reviews*, 103276.
- Suriano, J., Mardonez, D., Mahoney, J. B., Mescua, J. F., Giambiagi, L. B., Kimbrough, D., & Lossada, A. (2017). Uplift sequence of the Andes at 30 S: Insights from sedimentology and U/Pb dating of synorogenic deposits. *Journal of South American Earth Sciences*, *75*, 11-34.
- Trumbull, R. B., Riller, U., Oncken, O., Scheuber, E., Munier, K., & Hongn, F. (2006). The time-space distribution of Cenozoic volcanism in the South-Central Andes: a new data compilation and some tectonic implications. In *The Andes* (pp. 29-43). Springer, Berlin, Heidelberg.
- Victor, P., Oncken, O., & Glodny, J. (2004). Uplift of the western Altiplano plateau: Evidence from the Precordillera between 20 and 21 S (northern Chile). *Tectonics*, *23*(4).
