# Supplementary material for: Growth of Neogene Andes linked to changes in plate convergence using high-resolution kinematic models
Source: Nat Commun. 2022 Mar 15;13:1339. doi: 10.1038/s41467-022-29055-4 (PMC8924272; doi:10.1038/s41467-022-29055-4)
Supplement: Supplementary file 3 — Description of Additional Supplementary Files [file 41467_2022_29055_MOESM3_ESM.pdf]

Description of Additional Supplementary Files

File name: Supplementary Data 1
Description: Finite rotations reconstructing the past position of the Nazca-Farallon (NZ-FA) plate relative to the South America (SA) plate, obtained through the plate circuit NZ-FA – Pacific (PA) – Antarctica (AN) – Nubia (NU) – SA. Each row is formatted as follows: age (Ma), longitude of the rotation pole (deg E), latitude of the rotation pole (deg N), rotation angle (deg), entries (Cxx, Cxy, Cxz, Cyy, Cyz, and Czz) of the covariance matrix (rad2). Finite rotations are interpolated at the times younger than 30 Ma in the South America/Nubia reconstruction of DeMets & Merkouriev, 2019, which adopts the astronomically-tuned GTS12 timescale (Ogg 2012).

File name: Supplementary Data 2
Description: Stage Euler vectors for the motion of the NZ-FA plate relative to SA. Each row is formatted as follows: stage initial time (Ma), stage final time (Ma), longitude of the Euler pole (deg E), latitude of the Euler pole (deg N), angular velocity (deg/Myr), entries (Cxx, Cxy, Cxz, Cyy, Cyz, and Czz) of the covariance matrix (rad2/Myr2).

File name: Supplementary Data 3-4
Description: Same as supplementary files 1-2, but accounting for the relative motion between East Antarctica (AN) and West AN – that is, obtained through the plate circuit NZ-FA – PA – West AN – East AN– NU – SA.

File name: Supplementary Data 5
Description: Finite rotations reconstructing the past position of the Pacific (PA) plate relative to the AN, upon noise mitigation on the finite rotations of Croon et al., 2008 through the REDBACK software (Iaffaldano et al., 2014). Each row is formatted as follows: age (Ma), longitude of the rotation pole (deg E), latitude of the rotation pole (deg N), rotation angle (deg), entries (Cxx, Cxy, Cxz, Cyy, Cyz, and Czz) of the covariance matrix (rad2). Parameter values and diagnostics utilised for the noisemitigation process are reported in Supplementary file 6 and Supplementary Figure 1.

File name: Supplementary Data 6
Description: Values of the Redback parameters used for mitigation of noise in the finite rotations of Croon et al., 2008, which reconstruct the past position of PA relative to AN.

File name: Supplementary Data 7-10
Description: Noise-mitigated, time-interpolated versions of the finite rotation reconstructing the past position of NZ relative to PA (SF7), PA relative to AN (SF8), AN relative to the Nubia (Nu) plate (SF9), West AN relative to East AN (SF10). Finite rotations are interpolated at the times younger than 30 Ma in the SA/NU reconstruction of DeMets & Merkouriev, 2019, which adopts the astronomically-tuned GTS12 timescale (Ogg 2012). Within each file, each row is formatted as follows: age (Ma), longitude of the rotation pole (deg E), latitude of the rotation pole (deg N), rotation angle (deg), entries (Cxx, Cxy, Cxz, Cyy, Cyz, and Czz) of the covariance matrix (rad2).
